# Fringing Electric Field Sensors for Anti-Attack at System-Level Protection

**DOI:** 10.3390/s18093034

**Published:** 2018-09-11

**Authors:** Xiang Gao, Yiqiang Zhao, Haocheng Ma

**Affiliations:** 1 School of Microelectronics, Tianjin University, Tianjin 300072, China; gx199391@163.com (X.G.); 18522921385@163.com (H.M.); 2 Tianjin Key Laboratory of Imaging and Sensing Microelectronic Technology, Tianjin 300072, China

**Keywords:** information security, system-level protection, fringing electric field sensor, sensor design

## Abstract

Information system security has been in the spotlight of individuals and governments in recent years. Integrated Circuits (ICs) function as the basic element of communication and information spreading, therefore they have become an important target for attackers. From this perspective, system-level protection to keep chips from being attacked is of vital importance. This paper proposes a novel method based on a fringing electric field (FEF) sensor to detect whether chips are dismantled from a printed circuit board (PCB) as system-level protection. The proposed method overcomes the shortcomings of existing techniques that can be only used in specific fields. After detecting a chip being dismantled from PCB, some protective measures like deleting key data can be implemented to be against attacking. Fringing electric field sensors are analyzed through simulation. By optimizing sensor’s patterns, areas and geometrical parameters, the methods that maximize sensitivity of fringing electric field sensors are put forward and illustrated. The simulation is also reproduced by an experiment to ensure that the method is feasible and reliable. The results of experiments are inspiring in that they prove that the sensor can work well for protection of chips and has the advantage of universal applicability, low cost and high reliability.

## 1. Introduction

Since the advent of the information era, people have been living in a world full of billions of computing systems, identifying, tracking and analyzing some of our intimate personal information including health, sleep, location, and network of friends [1]. However, as people are in pursuit of high performance and high speed, national information security and individual privacy security must be paid more attention. Information leakage has become one of the most pivotal risks in information society where sensitive confidential information such as personal data, company and even military intelligence are stored in electronic devices. Chips serve as the core component of information storage and communication, and they have become a pivotal source of information interception and data analysis for attackers. Some means of attacks such as invasive attacks, semi-invasive attacks and non-invasive attacks have been researched in the last several years. Corresponding countermeasures have also been developed by researchers all over the world [2,3,4,5]. However, existing protective techniques mainly focus on chip-level protection and few studies pay attention to system-level protection as primary safeguards.

It is important to detect whether a chip has been dismantled. If the chip is on PCB, it will be much more difficult for attackers to obtain confidential data or implement attacking. Chips that are not dismantled from PCB will make invasive attacks and semi-invasive attacks like optical emission analysis much harder [5]. Memory modification attacks were actively used to circumvent the security in microcontrollers. The chip MSP430F112 was dismantled and an infrared laser diode was used to irradiate the chip’s backside. After an optical fault masking attack, power supply of MSP430F112 was reduced to 2.5 V and embedded Flash and EEPROM (electrically erasable programmable read only memory)’s operations of writing and erasing were disabled, which offered the possibility of partial reverse engineering for chips by finding active locations [6]. For the self-help financial equipment for example ATMs, the password keyboard is an integral part. The password entered by the user is closely related to the key stored in the chip of a password keyboard. Attackers firstly dismantle the chip and then implement semi-invasive attack or invasive attack to acquire a key in the chip. That is a major property threat for everyone. For memory chips, if they are dismantled, the program and data in memories will be read out easily. Detecting dismantling chips from PCB is of crucial importance; however, current research mainly focuses on a protective manner in chips by integrating sensors with different functions. Detecting whether chips are dismantled can serve as the first line of defense. For the configuration chip of Field Programmable Gate Array (FPGA), if FPGA is dismantled and replaced by a programming device, its configuration program is easy to be read out. It is crucial to protect radio frequency identification devices’ (RFIDs) electronic tags from being dismantled to prevent them from being reused. It can be seen that detecting whether chips are dismantled from PCB is of great significance. When chips are dismantled, electronic systems should immediately recognize the attack and then take some defensive measures like deleting chip’s data to ensure confidentiality and integrity of sensitive information.

System-level protection is imperative, however, up to now, there have been no universally applicable methods to detect whether chips are dismantled from PCB as system-level protection and little relevant research has been done. Current methods can be classified into three classes: the first was using a tiny switch under a chip. Once the chip was dismantled, the switch was off, then the attack can be detected. However, for Surface Mounted Devices (SMD), it was really difficult to find such a small and thin switch that can be placed between the chip and PCB [7]; the second was taking advantage of a chip’s two pins and metal wire on PCB to form a loop. Once the chip was not on the PCB, the loop was no longer existing. Utilizing this principle, dismantling chips can be detected. However, for the majority of memories or FPGAs, there were no extra pins to use. In addition, the method will be invalid once the pins connected to the PCB are short-circuited [8]; the third was using laser sensors to detect whether the chip was on PCB. However, this method incurs prohibitive costs and is a tedious size, which can be only applied in specialized fields [9].

In consideration of the above approaches’ limitations, a system-level protection approach based on fringing electric field sensors are proposed to detect whether chips are dismantled from PCB or not. The FEF (fringing electric field) sensor can be combined with other anti-attack measures to form an integrated defense system. Chips that need to be protected can be packaged together with battery and low-power anti-attack chips, which have the function of capacitance detection of the fringing electric field sensors. It also acts as a security package, active shield, temperature detector and light detector. This will make sure that, even though power is off, a protection system can remain effective. The fringing electric field sensor is difficult to be analyzed due to its nonlinear feature and an extant model can only apply to ideal situations [10]. Therefore, in this paper, the conclusions are made on the basis of a simulation, and an experiment corresponding to the simulation is done to ensure the accuracy of the conclusions. For the purpose of improving a sensor’s sensitivity, a sensor’s patterns, areas and geometrical parameters were optimized to find the best structure. Finally, for a chip to be protected, the method of determining the sensor’s parameters to maximize a sensor’s sensitivity for easier measurement and signal conditioning was put forward. The experimental results are encouraging and certify that the proposed sensor provides an effective way to protect chips at the application of system-level protection.

The remainder of the paper is structured as follows. The theory of a fringing electric field sensor is described in Section 2. The sensor is modeled and simulation is done to find a sensor parameters’ influence on sensor’s sensitivity in Section 3. In Section 4, capacitance measurement circuit, experimental results and the analysis of experimental results are presented. The method that maximizes a sensor’s sensitivity is also put forward. In Section 5, the conclusions for this paper are summarized.

## 2. Theory of a Fringing Electric Field Sensor

Capacitive sensors have been used in a large variety of applications since they have high sensitivity and good immunity to temperature. At present, the most popular capacitive sensors are parallel-plate-type capacitors. The sensor forms one plate and the object to be measured forms the other plate. However, parallel-plate-type capacitors have some weaknesses: the object to be measured should be a conductor; the sensor can not be used in applications when one part is inaccessible, for example, the part is rotating or has a special coating. Due to these limitations, another type of capacitive sensor called a fringing electric field sensor was developed. Noltingk first proposed a fringing electric field sensor based on the principle of fringe capacitance [11]. The FEF sensor allows non-contact measurements for reason that electrodes of the sensor are completely isolated from the analyte, which is its most significant advantage over parallel-plate-type capacitors. Non-contact measurements with FEF sensors provide reliable information about material’s characteristic and property [12]. Due to possessing these features, the FEF sensor is used in many fields, for example, for soil moisture measurement, making changes in a material’s dielectrics, in motion sensors, to improve a material’s properties and to measure the moisture content of agricultural commodities [13,14,15]. The FEF sensors’ performance is largely impacted by their penetration depth, signal strength, measurement sensitivity and linearity, which are determined by factors such as sensor’s pattern, area and geometrical parameters [16].

**Figure 1:**
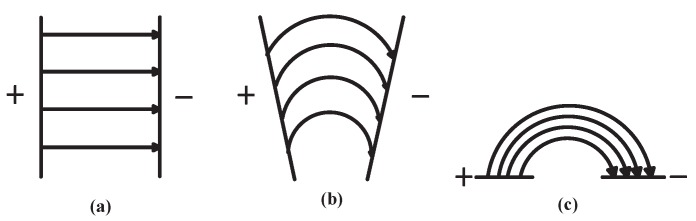
(**a**) is a parallel-plate capacitor; (**a**)’s electrodes are gradually opened up turning into (**b**); finally, it becomes (**c**), the fringing electric field sensor.

Figure 1 shows the forming process of a fringing electric field sensor. A FEF sensor can be imaged as putting a parallel-plate-type capacitors’ two plates in a coplanar arrangement as Figure 1. Electric field lines generate from one electrode and terminate on the other electrode. The electric field lines close to the surface of sensor penetrate the object to be tested on the sensor. The distribution of electrical parameters can be used to obtain information about the analyte’s physical properties [11]. When an object approaches the FEF sensor, it influences the distribution of an electric field line, the capacitance between two electrodes will change. The analyte does not need to get in touch with the sensor, so FEF sensors have the advantage of non-contact and non-destructive features.

On the basis of this theory, the FEF sensor with PCB technology can be designed and manufactured under the chip. The chip to be protected is located on the sensor. The capacitance of sensor when the chip is on PCB is *C*${}_{1}$, the capacitance of sensor when the chip is dismantled from PCB is *C*${}_{2}$. The capacitance’s change after attack is Δ*C*:

(1) $$\Delta C\;=\;C_{1}-C_{2}.$$

The change in capacitance can be recognized as attacking on the chip and can be used to trigger an alarm signal. Then, protective measures can be taken to prevent leakage of information.

**Figure 2:**
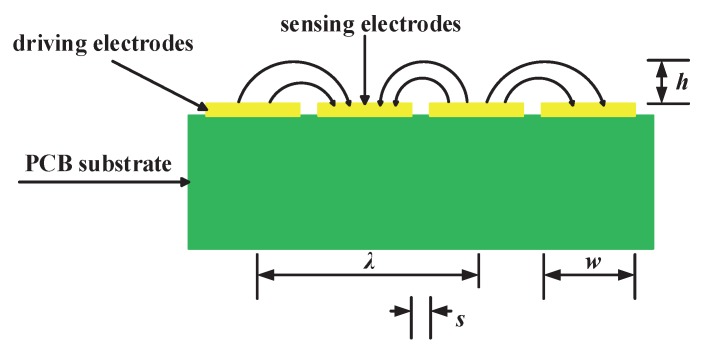
Structure and electric field lines of fringing electric field sensors.

Figure 2 shows structure and electric field lines of the sensor. Electric field lines generate from driving electrodes and terminate at sensing electrodes, air gaps are maintained between the electrodes. Penetration depth *h* of the fringing electric field sensors’ electric field line is proportional to the length of λ [17]. Penetration depth represents measurement range of the sensor. Penetration depth defines a physical quantity, which decay rate of a sensor’s electric field intensity accelerates as the distance between the object to be measured and the sensor increases [18]. Measurement range of a fringing electric field sensor is proportional to penetration depth. When penetration depth increases, the measurement range of a sensor increases correspondingly, but a sensor’s signal strength will decrease. If penetration depth decreases, the measurement range of fringing electric field sensor will diminish. However, a sensor’s signal strength is greater. λ is the distance between the centerlines of the two adjacent driving or sensing electrodes. λ is determined by *w* (the width of electrodes) and *s* (the spacing between two contiguous driving electrodes and sensing electrodes), their influence on fringing electric field sensor will be analyzed in the following section.

**Figure 3:**
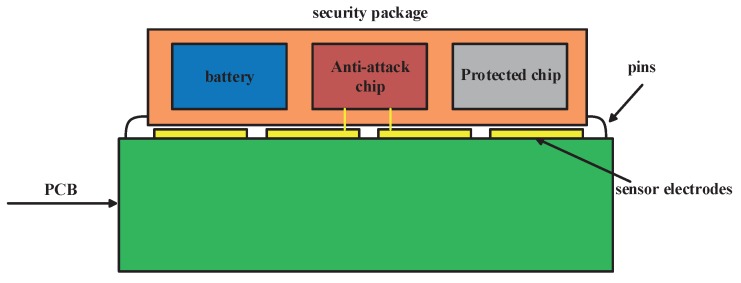
Safe protection system.

The FEF sensor, together with other anti-attack means, can form a reliable defense system. Figure 3 shows the structure of a safe protection system. The protected chip is packaged together with a battery and anti-attack chip. The battery is used to ensure that the protection system can still work well even if power is off. The anti-attack chip includes a capacitance measuring circuit that detects FEF sensor’s capacitance change to identify whether the chip is being attacked. It can also have many functions to resist invasive attacks and non-invasive attacks. Anti-attack chip can integrate various sensors, such as a temperature sensor to resist temperature attack, an optical sensor to resist optical attacks, an active shield to resist a FIB (Focused Ion Beam) attack, and external voltage detection to resist a voltage attack. To avoid discharging the battery, which will make protection system fail, it should also have the function of detecting battery capacity. When battery capacity is below the threshold value, it will send out an alarm signal, and then key data in a protected chip will be deleted. The package body is made of ceramic and a ceramic substrate is in the packaging body. The battery, anti-attack chip and protected chip are placed on the substrate and packaged with COB (Chips on Board). For a capacitance measuring circuit, it will add two extra pins to connect the FEF sensor’s two electrodes. However, if an anti-attack chip is comprised of a temperature sensor to resist temperature attack, it will add no extra pins. The increased numbers of pins depend on an anti-attack chip’s function. The extra power consumption of capacitance measuring circuit is 20 μA and power consumption of other anti-attack sensors like temperature sensor and light sensor are less than 20 μA. When battery capacity is below threshold value, a user should charge the battery. When battery capacity continues to decrease, this means that the chip is being attacked and the chip will generate an alarm signal. When the anti-attack chip detects attack, it will delete confidential data or take other measures to keep the chip safe. Other measures include an exothermal energy release layer for microchip transience. A proportional combination of self-assembled CuO/Al nanothermite and Napalm-B as gelling agent was used to develop a spinable nanothermite film onto the surface of a micro-chip. The coated energy release layer is ignitable using a microfabricated heater or an electric spark to melt the surface of the underlying substrate and any surface-bound microdevices and electronic feature [19]. Another measure is using physical methods to destroy the chip. A transience mechanism for silicon microchips via low-temperature postprocessing steps that transform almost any electronic or MEMS (micro-electro-mechanical system) substrate chips into transient ones. Triggered chip transience is achieved by the incorporation of a distributed, thermally-activated expanding material on the chip backside. When heated at 160 ${}^{\circ }$C, the expanding material produces massive chip cleavage mechanically shattering the chip into a heap of silicon dust [20]. In a word, after detecting attack, deleting key data or destroying chips are countermeasures to prevent attackers getting important information.

## 3. Design and Simulation

The design of a fringing electric field sensor is of the essence: design and simulation can help find the ways to maximize sensor’s sensitivity so that a subsequent signal processing circuit can easily detect sensor’s capacitance change when the chip is attacked. The sensor consists of two electrodes and a substrate. Electrodes are made of copper and the substrate is PCB. The fringing electric field sensor’s electrodes are composed of copper whose thickness ranges from 17.78–71.12 μm depending on the manufacturing process [21]. PCB is the support body of electronic components and the carrier of electrical connection. Generally, the substrate of PCB is composed of a special material named FR-4 (woven glass and epoxy), which is featured with stable electrical insulation, good flatness, high mechanical properties and high heat resistance. The thickness of electrodes is 35 μm and the thickness of PCB substrate is 1.57 mm in this paper.

**Figure 4:**
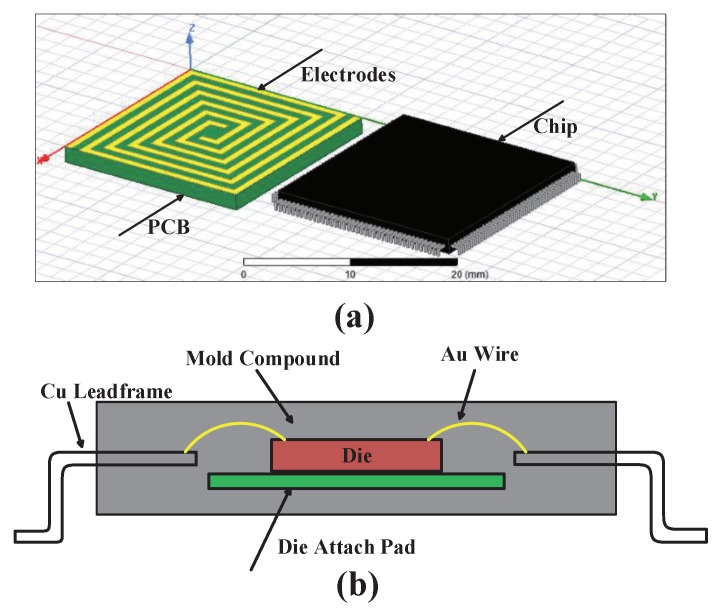
(**a**) is a model of simulation in Ansoft Maxwell 3D; (**b**) is a package model.

The fringing electric field sensor was modeled and analyzed with the finite element method (FEM). The FEM is often used in analyzing and computing of fluid mechanics, electromagnetism and structural mechanics [22]. Ansoft Maxwell 3D (a part of ANSYS Electromagnetics Suite 18.0, ANSYS, Pittsburgh, PA, USA) with the capability to solve the magnetic or electric field distribution in a finite region is one of the most famous commercial low frequency electromagnetic finite element software programs [23]. Ansoft Maxwell 3D was applied to establish a three-dimensional (3D) model to analyze how the sensor’s structures and parameters influence the sensor’s capacitance change. The purpose of simulation was to find out what factors have influence on sensor’s capacitance change. The parameters of FEF sensor in simulation is coincident with the fact and the parameters of the chip to be protected should be determined. SMD is the most popular craft in modern electronic assembly industry and almost all kinds of chips can be packaged with SMD, so research on chips packaged with SMD was done in this paper. The model of chip in simulation was a TQFP144 (thin quad flat package 144 pins) whose length was 2 cm, the width was 2 cm and the thickness was 1.5 mm, the distance between the chip and PCB was 0.1 mm. The sensor’s capacitance before and after the chip was off the PCB was generated through simulation. Figure 4 shows a model of simulation in Ansoft Maxwell 3D and the chip package model. The die is composed of Si and mold compound is composed of epoxy whose dielectric constant is 3.6 to imitate plastic package in simulation. The option of Length Based Refinement was applied in mesh operation and Length of Elements is set to 1 mm. Changes in the mesh did not significantly alter the simulation results.

### 3.1. Influence of the Fringing Electric Field Sensor’s Pattern

**Figure 5:**
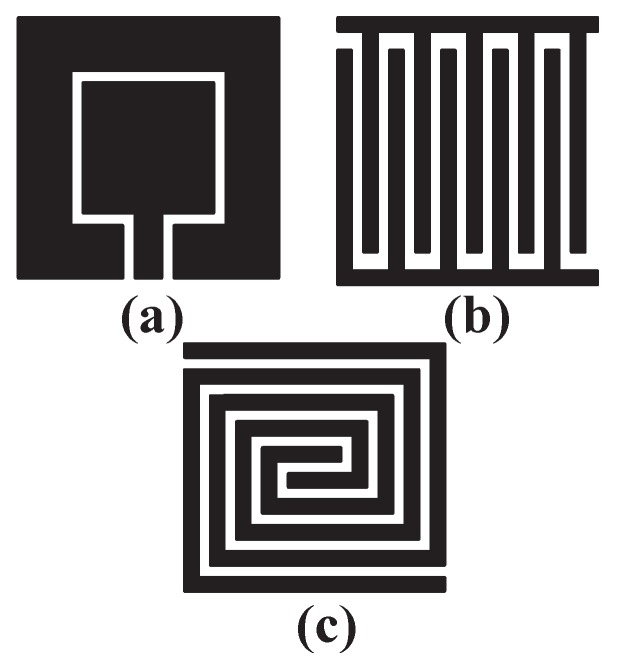
(**a**) square-shaped pattern; (**b**) interdigital pattern; (**c**) spiral pattern.

**Figure 6:**
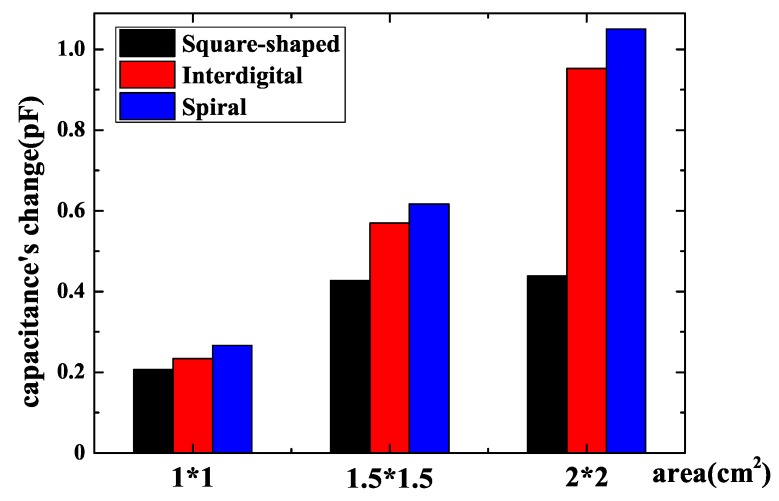
Change of the sensor’s capacitance with different patterns and different areas.

Different sensor’s patterns have different influences on capacitance’s change. In this step, the goal is to find the relationship between the sensor’s pattern and capacitance’s change. As the change of sensor’s capacitance becomes greater, it is easier for a subsequent circuit to recognize this change and improve the reliability of system. Figure 5 shows the schematic drawings of three different patterns: they are square-shaped pattern, interdigital pattern and spiral pattern, which have the same area, the same electrodes’ width and the same spacing between two electrodes. Square-shaped pattern, interdigital pattern and spiral pattern sensors were widely used and researched in previous papers [24,25,26]. Models were established in these three patterns and the results of different patterns with different areas in simulation are presented in Figure 6. The parameters of FEF sensors in Figure 6 are shown in Table 1. It can be observed that a square-shaped pattern causes the least variation of capacitance, interdigital and spiral patterns are obviously better than square-shaped pattern, while a spiral pattern is a little better than an interdigitated pattern. As a result, a spiral pattern fringing electric field sensor that has the best performance is a good option.

### 3.2. Influence of a Fringing Electric Field Sensor’s Area

After choosing the best pattern for a sensor, a sensor’s area should be taken into consideration. Since the chip is packaged with SMD, the sensor’s area can only be the same with the chip or smaller than the chip for fear that a sensor’s electrodes make contact with the chip’s pins. If the fringing electric field sensor’s area decreases, it is better for PCB routing. However, it may make the capacitance’s change smaller and make it difficult to measure. Simulation of square-shaped, interdigital and spiral pattern fringing electric field sensor with various areas of 2 cm × 2 cm, 1.5 cm × 1.5 cm and 1 cm × 1 cm was done and the result of simulation is presented in Figure 6. One can observe that the sensor’s capacitance changes significantly with a sensor’s area. The capacitance’s change becomes greater when sensor’s area becomes larger. As a result, to acquire maximum capacitance’s change, a larger sensor whose size is equal to the chip’s size should be manufactured. A larger sensor’s area means less area for routing. There is a trade-off between sensor’s area and capacitance’s change.

### 3.3. Influence of Fringing Electric Field Sensor’s Geometrical Parameters

It is important to choose the best geometrical parameters so as to maximize its sensitivity, the variation of the sensor’s capacitance. Like what has been discussed before, the best pattern and area were determined. Geometrical parameters like electrodes’ width and spacing between electrodes should be taken into consideration.

**Figure 7:**
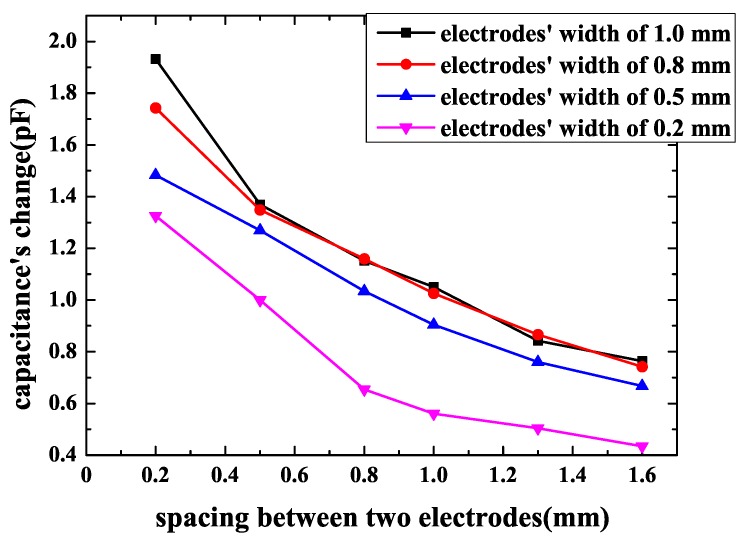
Capacitance’s change of the spiral pattern sensor with electrodes’ spacing.

In order to find out the relationship between spacing and capacitance’s change of sensors, the electrodes’ width was fixed at 1 mm, 0.8 mm, 0.5 mm and 0.2 mm, and the spacing between driving electrodes and sensing electrodes ranged from 0.2–1.6 mm. The sensor’s pattern was spiral and the area was 2 cm × 2 cm—these are the best structural properties based on the theories that were summarized earlier. The result is shown in Figure 7. It can be noted that capacitance’s change decreases as spacing increases. For example, when an electrodes’ width is 1mm, spacing varies from 0.2–1.6 mm, and capacitance’s change decreases from 1.932–0.7643 pF. This means that, in order to obtain larger capacitance change, spacing between two electrodes should be as small as possible.

**Figure 8:**
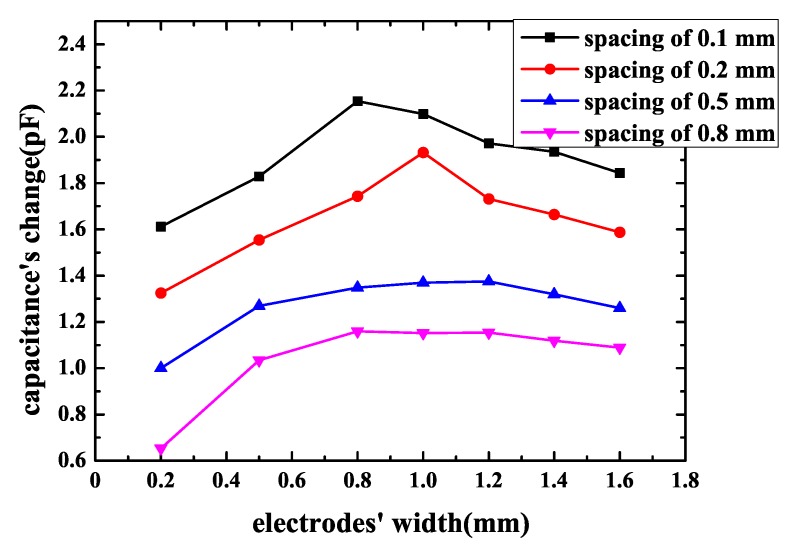
Capacitance’s change of the spiral pattern sensor with electrodes’ width.

The influence of an electrodes’ width was simulated when spacing was fixed. The sensor’s pattern was spiral and the area of sensor was 2 cm × 2 cm. The sensor’s spacing was 0.1 mm, 0.2 mm, 0.5 mm, 0.8 mm and electrodes’ width ranged from 0.2–1.6 mm when spacing is fixed. The simulation results are shown in Figure 8. It can be seen that, for a certain spacing, the change of fringing electric field sensor’s capacitance varies with electrodes width’s change and when electrodes’ width reaches some point, sensor’s capacitance change comes to a maximum value. The parameters of FEF sensors in Figure 7 and Figure 8 are shown in Table 2.

## 4. Experimental Results and Discussion

### 4.1. Experiment Process and Results

**Figure 9:**
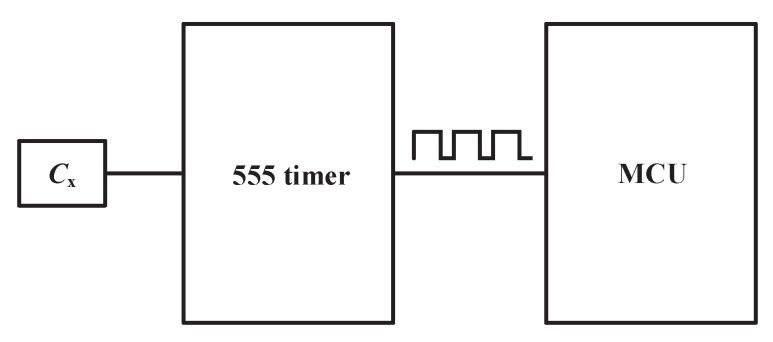
Measurement circuit of a fringing electric field sensor.

A low-cost method that can fulfill the requirements of the application and detection accuracy was adopted. The measurement circuit is showed in Figure 9. The system consists of two major chips, 555 timer and Microcontroller Unit (MCU). *C${}_{x}$* is the capacitance to be measured. When power is on, the output of 555 timer is a square-wave signal. The period of the square-wave signal *T* is in proportion to *C${}_{x}$*. The working process of the circuit can be divided into two parts: (a) first, when the circuit is powered, the capacitor *C${}_{x}$* has not been charged. The voltage of the capacitor *U${}_{x}$* is lower than reference low voltage *U${}_{L}$*, the output is high. Then, *U${}_{x}$* is charged by power supply through *R*${}_{1}$ and *R*${}_{2}$, so *U${}_{x}$* increases exponentially. When *U${}_{x}$* rises to reference high voltage *U${}_{H}$*, the output is low, and the capacitor starts to discharge. The time of *U${}_{x}$* rising from *U${}_{L}$* to *U${}_{H}$* is *T*${}_{1}$:

(2) $$T_{1}=\left(R_{1}+R_{2}\right)*C_{x}*\ln2,$$

(b) Second, Capacitor *C${}_{x}$* discharges by *R*${}_{2}$, so *U${}_{x}$* declines. When *U${}_{x}$* drops to *U${}_{L}$*, the output is high. The time of *U${}_{x}$* droping from *U${}_{H}$* to *U${}_{L}$* is *T*${}_{2}$:

(3) $$T_{2}=R_{2}*C_{x}*\ln2.$$

Then, the circuit repeats process (a) and (b). Output of the 555 timer will generate a square wave, and the period of output is *T*:

(4) $$T=T_{1}+T_{2}=\left(R_{1}+2R_{2}\right)*C_{x}*\ln2.$$

**Figure 10:**
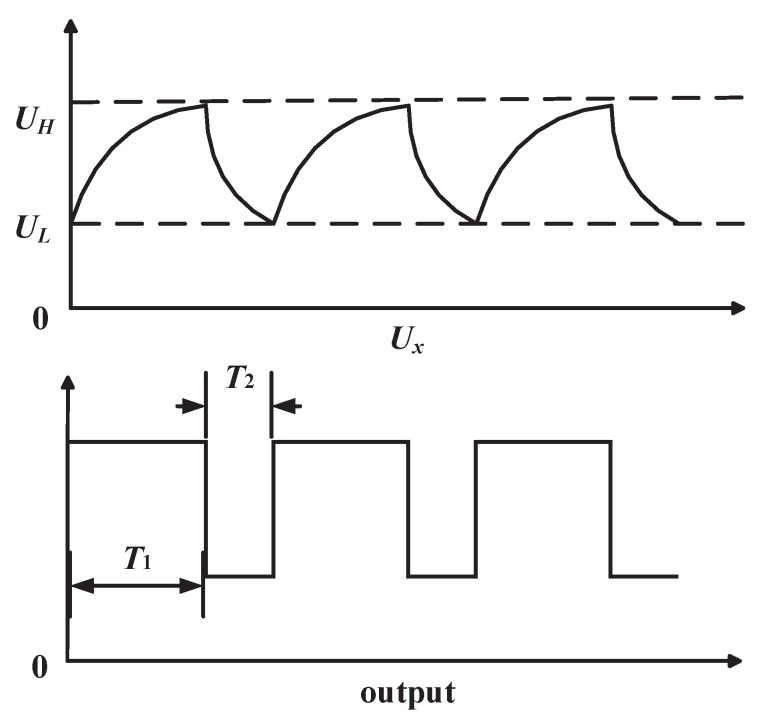
capacitance to period conversion circuit’s waveform.

The output wave is shown in Figure 10.

The output of the 555 timer is directly connected to a microcontroller’s T0 port and microcontroller’s timer0 counts for T0. By counting for a T0 port in a known fixed time and checking the count value, the period *T* can be computed. As the value of *T*, *R*${}_{1}$ and *R*${}_{2}$ is known, and the capacitance to be measured is easy to work out according to Equation (4).

**Figure 11:**
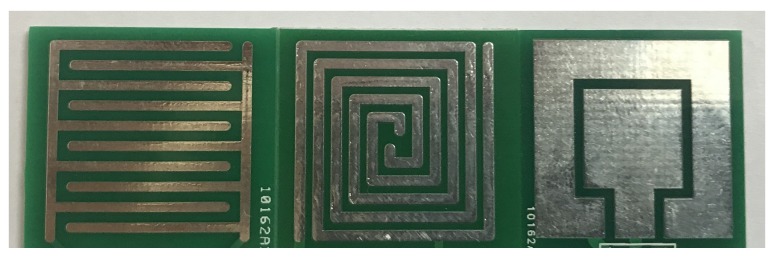
Photograph of prototype sensors.

In order to verify the conclusions from simulation, fringing electric field sensors were fabricated. A photograph of prototype sensors is shown in Figure 11. All of these capacitive sensors were manufactured using a two-layer PCB process. The capacitive sensors’ pattern, electrodes’ width and spacing were consistent with simulation to ensure its credibility. Firstly, FEF sensor was connected to a 555 timer and the chip to be protected was placed on the FEF sensor. The output of a 555 timer generated square wave and a microcontroller counted for it in a fixed time. The count value was displayed by a microcontroller’s output pins. According to Equation (4), FEF sensor’s capacitance when the chip was on the sensor was computed. Then, the chip to be protected was taken off of the FEF sensor and the sensor’s capacitance was computed in the same way. Finally, a sensor’s capacitance change can be easily obtained.

**Figure 12:**
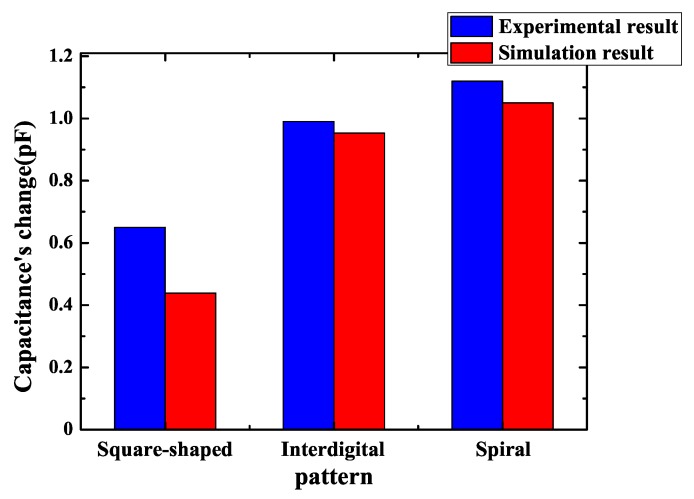
Experimental results of sensor capacitance’s change with different patterns.

So as to ensure the consistency between simulation and experiment, all parameters of the sensors were equal to models of simulation. Firstly, three fringing electric field sensors of different patterns in Figure 5 were manufactured and the capacitance was measured using the circuit of Figure 9. The result of measurement is shown in Figure 12. The parameters of FEF sensors in Figure 12 are shown in Table 3. The capacitance’s change of a square-shaped pattern, interdigital pattern and spiral pattern in experiments are 0.65 pF, 0.99 pF and 1.12 pF. It can be seen that the results corresponded to simulation. The spiral pattern is the best structure among three patterns, while the interdigital pattern are better than a square-shaped pattern. Experimental results show that the spiral pattern is the most suitable sensor pattern for protecting the chip at system-level protection.

**Figure 13:**
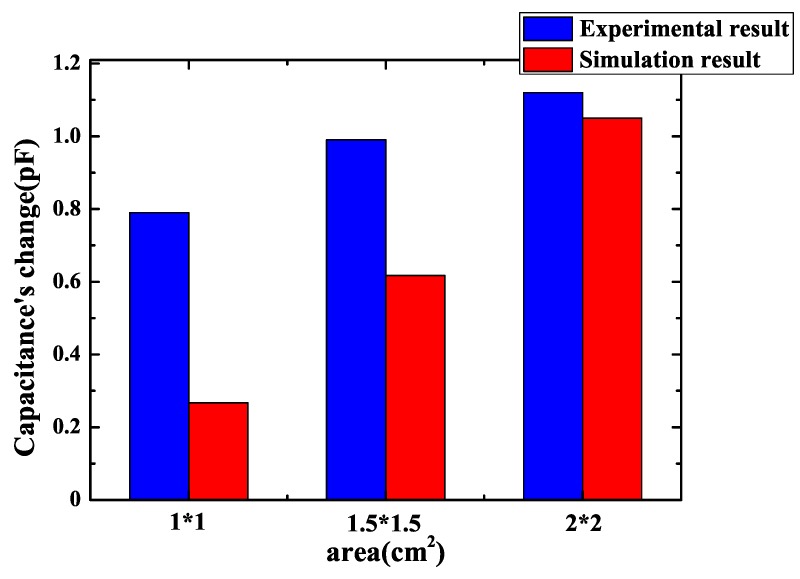
Experimental results of a spiral pattern sensor capacitance’s change with different areas.

As the best pattern for sensors was determined, experiments with different sensors’ areas were carried out. Measurement of a spiral pattern fringing electric field sensor with an area of 2 cm × 2 cm, 1.5 cm × 1.5 cm and 1 cm × 1 cm consistent with simulation was done and the results are presented in Figure 13. The parameters of FEF sensors in Figure 13 are shown in Table 4. The capacitance’s change of these sensors with three different areas in experiments are respectively 0.79 pF, 0.99 pF and 1.12 pF. It is shown that the capacitance’s change gets larger when a sensor’s area gets larger. Consequently, in order to maximize capacitance’s change, the area of a sensor should be equal to the chip’s area. However, as what has been discussed, a large sensor means area for routing is reduced. If area for routing is essential, a small sensor can be made as long as capacitance change of a small sensor can be distinguished by a subsequent circuit.

**Figure 14:**
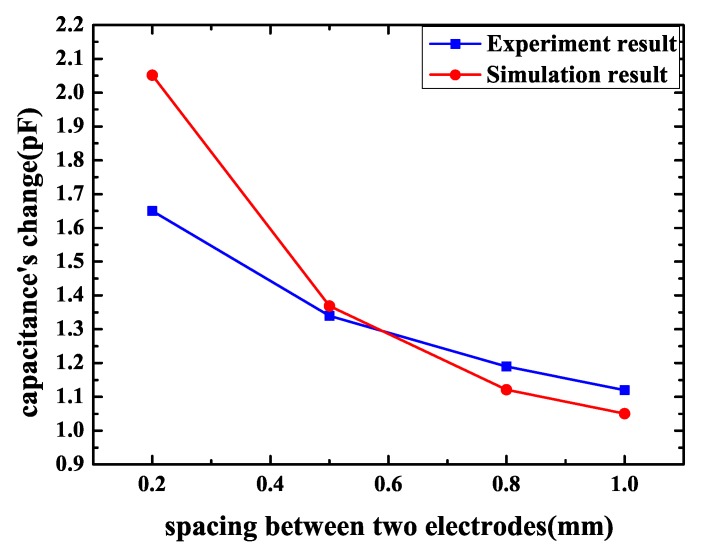
Experimental results of a spiral pattern sensor capacitance’s change with electrodes’ spacing.

As what has been discussed before, the best pattern and area were determined. Geometrical parameters’ influence should also be confirmed. Firstly, the influence of spacing between two electrodes was measured. The parameters were equal to simulation that the electrodes’ width was 1 mm and the spacing between two electrodes ranged from 0.2–1.0 mm. The sensor’s pattern was spiral and the area was equal to the chip of 2 cm × 2 cm. The result is shown in Figure 14. The parameters of FEF sensors in Figure 14 are shown in Table 5. When spacing becomes smaller, capacitance’s change increases. This phenomenon is consistent with simulation. When spacing changes from 0.2–1.0 mm, capacitance’s change increases by 0.53 pF. Thus, small spacing between two electrodes should be a better choice for bigger capacitance’s change.

**Figure 15:**
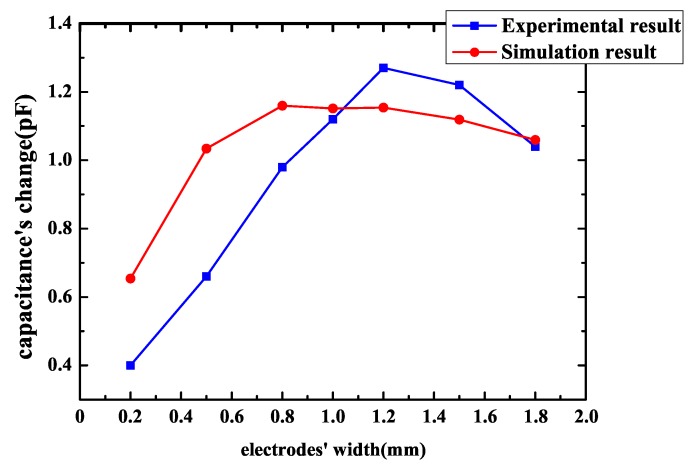
Experimental results of spiral pattern sensor capacitance’s change with electrodes’ width.

Then, the influence of electrodes’ width is measured. The spacing is 0.8 mm and electrodes’ width varies from 0.2–1.8 mm. The result of experiment is shown in Figure 15. The parameters of FEF sensors in Figure 15 are shown in Table 6. It can be noticed that, as electrodes’ width gets bigger, a sensor’s capacitance change gets bigger at first, when electrodes’ width reaches the best width, capacitance’s change reaches a maximum value, then as width gets bigger, capacitance’s change gets smaller. The best electrodes’ width for the chip in this paper is 1.2 mm. For the anti-attack fringing electric field sensor, the capacitance is not just determined by the sensor’s pattern and size, it is also influenced by the geometrical parameters of the sensor to a great extent. On the whole, the results of measurement correspond to the simulation models’ predictions pretty well.

### 4.2. Discussion

On the whole, the experimental results are in accordance with simulation results. As sensor’s patterns, areas and geometrical parameters change, experimental results and simulation results present the same trend. However, the concrete value of capacitance’s change is different. The reason is that a simulation model’s component of the chip is too simple. The real chip’s component is much more complex, so a real chip’s dielectric constant is different from a simulation model’s dielectric constant. Furthermore, when electrodes’ width and spacing are below 0.5 mm, simulation accuracy drops, so there are distinct differences between simulation results and experimental results. There are also instrumental errors and sensors manufacturing errors may also have been present in the experiment.

According to simulation and experiments, the optimal design method is as summarized below. Spiral pattern is the best because the overall sensor sensitivity is proportional to the effective electrode length [25]. The spiral pattern was designed in order to maximize its effective electrode length. For sensors with the same area, the sensor that has a complex pattern has greater effective electrode length and consequently has a much higher sensitivity. Among these three patterns, the spiral pattern sensor has the biggest effective electrode length and has the biggest capacitance’s change. Consequently, a complex pattern with long effective electrode length is a good choice.

The reason for a larger sensor with larger capacitance’s change is that the sensor’s sensitivity is proportional to both effective electrode length and the electrode area [25]. For the sensor with the same electrodes’ width and spacing, when the sensor’s area get bigger, its electrodes area get bigger and the sensor can contain more electrodes, which means it has longer effective electrode length. As a result, as what is shown in Figure 13, the sensor’s capacitance change is proportional to the sensor’s area. The biggest possible area of sensor is equal to the area of the chip to be protected.

For a certain electrodes’ width, the capacitance’s change of sensor becomes greater as spacing between two electrodes gets smaller. The reason is that when spacing decreases, total capacitance of the FEF sensor increases and the capacitance’s change caused by the chip to be protected becomes larger accordingly. According to simulation and measurement, spacing’s influence is more obvious and important than electrode width’s influence. When electrodes’ width is fixed and spacing decreases from 1–0.2 mm, capacitance’s change increases distinctly. To improve sensor’s sensitivity, spacing should be small. However, for a certain spacing between two electrodes, the sensor has the best electrodes’ width. The reason is that, as mentioned in Section 2, penetration depth of the fringing electric field sensors’ electric field line is proportional to the length of λ, which is the distance between the centerlines of the two adjacent driving or sensing electrodes. Penetration depth has an influence on sensor’s measurement range and capacitance’s magnitude, and, when penetration depth increases, the electric field line can reach a farther distance; therefore, the FEF sensor’s measurement range increases [16]. However, penetration depth’s incremental results in sensor’s capacitance magnitude’s decreases. As a result, capacitance’s change caused by the chip to be protected may reduce. According to what has been discussed above, there is a best penetration depth, while penetration depth is dependent on the distance between the centerline of the two same adjacent electrodes, which is equal to two times the sum of electrode width and electrode spacing. Penetration depth is determined by the sum of electrode width and electrode spacing. A best electrode width exists. In other words, the best sum of electrode width and electrode spacing exists. As a consequence, for a certain spacing, the sensor has the best electrodes’ width. The best electrodes’ width should be determined based on the actual situation. However, spacing’s impact is significant and it is greater than the impact of electrodes’ width. As long as spacing decreases, capacitance change of the sensor increases. The effect of electrodes’ width is less pronounced, and the best electrodes’ width is when spacing is constant.

Generally, errors in fringing electric field sensors measurement system come from sources such as the environment conditions, accuracy of sensor manufacturing and the instrumentation errors. However, measurement results were quite stable and reliable. It can be seen from simulation and measurement that the fringing electric field sensor is fit and can be useful in an information security field. It can be used to accurately detect whether a chip is dismantled from PCB at system-level protection. In this paper, the qualitative analysis was mainly made because, as mentioned in Section 1, a fringing electric field sensor is difficult to be analyzed due to its nonlinear feature. Research was mostly done through simulation and experiments at present. Quantitative analysis is the goal of future research.

## 5. Conclusions

Information security gets more and more vital and much attention has been paid to it in recent years. Chips serve as the fundamental elements of the information technology industry and become attackers’ targets. This paper investigated and discussed the feasibility of using the fringing electric field sensor to detect whether a chip is dismantled as system-level protection. After detecting an attack, some protective measures like deleting key data can be used for attack resistance. Simulations were done to analyze fringing electric field sensor’s performance. Experiments were also done to evaluate and verify the sensor’s reliability. The method of designing a sensor’s pattern, area and geometrical parameters to maximize sensor’s sensitivity was put forward and illustrated. In conclusion, according to simulations and experiments, the proposed sensor in this paper will be an effective anti-attack option.

## Figures and Tables

**Table 1:** Parameters of FEF (fringing electric field) sensors in Figure 6.

Pattern	Area (cm${}^{2}$)	Electrode Spacing (mm)	Electrode Width (mm)	Number of Fingers or Loops
Square-shaped	1 × 1	1	N/A	N/A
Square-shaped	1.5 × 1.5	1	N/A	N/A
Square-shaped	2 × 2	1	N/A	N/A
Interdigitated	1 × 1	1	1	5
Interdigitated	1.5 × 1.5	1	1	8
Interdigitated	2 × 2	1	1	10
Spiral	1 × 1	1	1	3
Spiral	1.5 × 1.5	1	1	4
Spiral	2 × 2	1	1	5

**Table 2:** Parameters of FEF (fringing electric field) sensors whose area are 2 cm × 2 cm and patterns are interdigital in Figure 7 and Figure 8.

		Electrode Width (mm)	0.2	0.5	0.8	1	1.2	1.4	1.6
	Number of Loops								
Electrode Spacing (mm)									
0.1			33	16	10	9	7	6	5
0.2			25	14	10	8	7	6	5
0.5			14	10	7	7	6	5	4
0.8			10	8	6	6	5	5	4
1			9	7	6	3	N/A	N/A	N/A
1.3			7	6	3	3	N/A	N/A	N/A
1.6			6	5	3	3	N/A	N/A	N/A

**Table 3:** Parameters of FEF (fringing electric field) sensors in Figure 12.

Pattern	Area (cm${}^{2}$)	Electrode Spacing (mm)	Electrode Width (mm)	Number of Fingers or Loops
Square-shaped	2 × 2	1	N/A	N/A
Interdigitated	2 × 2	1	1	10
Spiral	2 × 2	1	1	5

**Table 4:** Parameters of FEF (fringing electric field) sensors in Figure 13.

Pattern	Area (cm${}^{2}$)	Electrode Spacing (mm)	Electrode Width (mm)	Number of Loops
Interdigitated	1 × 1	1	1	3
Interdigitated	1.5 × 1.5	1	1	4
Interdigitated	2 × 2	1	1	5

**Table 5:** Parameters of FEF (fringing electric field) sensors in Figure 14.

Pattern	Area (cm${}^{2}$)	Electrode Spacing (mm)	Electrode Width (mm)	Number of Loops
Interdigitated	2 × 2	0.2	1	8
Interdigitated	2 × 2	0.5	1	7
Interdigitated	2 × 2	0.8	1	6
Interdigitated	2 × 2	1	1	5

**Table 6:** Parameters of FEF (fringing electric field) sensors in Figure 15.

Pattern	Area (cm${}^{2}$)	Electrode Spacing (mm)	Electrode Width (mm)	Number of Loops
Interdigitated	2 × 2	0.8	0.2	10
Interdigitated	2 × 2	0.8	0.5	8
Interdigitated	2 × 2	0.8	0.8	6
Interdigitated	2 × 2	0.8	1	6
Interdigitated	2 × 2	0.8	1.2	5
Interdigitated	2 × 2	0.8	1.5	5
Interdigitated	2 × 2	0.8	1.8	4
